# A Study on the Microstructure Regulation Effect of Niobium Doping on LiNi_0.88_Co_0.05_Mn_0.07_O_2_ and the Electrochemical Performance of the Composite Material under High Voltage

**DOI:** 10.3390/ma17092127

**Published:** 2024-04-30

**Authors:** Xinrui Xu, Junjie Liu, Bo Wang, Jiaqi Wang, Yunchang Wang, Weisong Meng, Feipeng Cai

**Affiliations:** Energy Research Institute, Qilu University of Technology (Shandong Academy of Sciences), Jinan 250014, China; xuxinrui0428@163.com (X.X.); wangb@sderi.cn (B.W.);

**Keywords:** lithium, high-nickel, ternary cathode material, Nb, doping, high voltage

## Abstract

High-nickel ternary materials are currently the most promising lithium battery cathode materials due to their development and application potential. Nevertheless, these materials encounter challenges like cation mixing, lattice oxygen loss, interfacial reactions, and microcracks. These issues are exacerbated at high voltages, compromising their cyclic stability and safety. In this study, we successfully prepared Nb^5+^-doped high-nickel ternary cathode materials via a high-temperature solid-phase method. We investigated the impact of Nb^5+^ doping on the microstructure and electrochemical properties of LiNi_0.88_Co_0.05_Mn_0.07_O_2_ ternary cathode materials by varying the amount of Nb_2_O_5_ added. The experimental results suggest that Nb^5+^ doping does not alter the crystal structure but modifies the particle morphology, yielding radially distributed, elongated, rod-like structures. This morphology effectively mitigates the anisotropic volume changes during cycling, thereby bolstering the material’s cyclic stability. The material exhibits a discharge capacity of 224.4 mAh g^−1^ at 0.1C and 200.3 mAh g^−1^ at 1C, within a voltage range of 2.7 V–4.5 V. Following 100 cycles at 1C, the capacity retention rate maintains a high level of 92.9%, highlighting the material’s remarkable capacity retention and cyclic stability under high-voltage conditions. The enhancement of cyclic stability is primarily due to the synergistic effects caused by Nb^5+^ doping. Nb^5+^ modifies the particle morphology, thereby mitigating the formation of microcracks. The formation of high-energy Nb-O bonds prevents oxygen precipitation at high voltages, minimizes the irreversibility of the H2–H3 phase transition, and thereby enhances the stability of the composite material at high voltages.

## 1. Introduction

In recent years, lithium-ion batteries have become crucial for generating renewable electricity from solar and wind sources, supporting sustainable development [1,2,3]. Lithium-ion batteries store renewable electrical energy that can be harnessed by electric vehicles, homes, and factories, reducing their dependence on fossil fuels [4,5,6,7]. Current research efforts are focused on enhancing the performance of lithium-ion batteries, aiming for higher energy density, superior safety, an extended cycle life, and cost reduction. The cathode materials play a pivotal role in determining the performance of lithium-ion batteries. Presently, the primary cathode materials for lithium-ion batteries include LiCoO_2_ [8], LiFePO_4_ [9], LiMn_2_O_4_ [10], and LiNi_x_Co_y_Mn_1–x–y_O_2_ [11,12,13]. LiCoO_2_ provides high energy density but is cost-intensive. LiFePO_4_ demonstrates relatively high energy density, an extended cycle life, and superior safety characteristics. Currently, LiFePO_4_ is the most widely utilized cathode material; nevertheless, its energy density has nearly reached its theoretical ceiling. The advancement of cathode materials with improved energy density, superior safety, and a prolonged cycle life is crucial for fulfilling industrial requirements.

The high-nickel ternary cathode material, known as Nickel Cobalt Manganese Lithium (NCM), is among the most promising candidates. As the nickel content increases, the capacity of the ternary cathode material also increases. When the nickel content exceeds 80%, the capacity of the material surpasses 200 mAh g**^−^**^1^ at 0.1C [14,15]. However, elevating the nickel content also poses several challenges. For example, the cation-mixing phenomenon becomes more prevalent as the nickel content increases [16]. The similarity in ionic radii between Ni^2+^ and Li^+^ ions makes complete oxidation of Ni^2+^ to Ni^3+^ challenging, even in a pure oxygen environment, during preparation. This leads to cation mixing between Ni^2+^ and Li^+^ ions. This mixing occurs throughout both material preparation and the charge–discharge process [17]. Cation mixing disrupts the deintercalation of Li^+^ ions, resulting in a reduction in the Coulomb efficiency and material capacity. Furthermore, during charging, Ni^2+^ oxidizes to Ni^3+^ and Ni^4+^, leading to a decrease in the ionic radius and interlayer structure stability, ultimately compromising the cyclic stability and safety [18]. The ternary cathode material experiences phase transitions throughout the charging and discharging process. Hexagonal phase transition from H2 to H3 occurs at higher voltages, with increasing significance above 4.2 V [19]. Lattice oxygen precipitation accompanies this transition. High-valence nickel ions migrate at high voltages due to Li/O vacancies, resulting in surface reconstruction and the formation of a rock salt phase, thereby affecting the capacity and cycle stability. Furthermore, high-valence Ni ions exhibit increased reactivity with the electrolyte, leading to structural damage [20]. Stabilizing high-nickel ternary materials under high-voltage conditions poses a significant challenge. Microcracks in secondary particles arise due to alterations in the crystal cell parameters during cycling [21,22]. Additionally, addressing concerns such as surface alkali residues and transition metal dissolution is crucial for maintaining the long-term stability of high-nickel NCM materials.

To ensure the durable and reliable performance of high-nickel NCM cathode electrode materials, surface-coating and bulk-doping techniques are utilized by researchers. Surface coating serves to stabilize the material’s surface structure, preventing interaction with the electrolyte, thus mitigating side reactions and bolstering cycle stability [23,24]. Investigators have examined the impact of coating with oxides, including MgO [25], CeO_2_ [26], Al_2_O_3_ [27], WO_3_ [28], and La_2_O_3_ [29], revealing that suitable oxide coatings effectively mitigate side reactions and boost material cycle stability. Bulk doping involves the introduction of elements to stabilize the material’s structure during cycling. Doping with elements such as Mg^2+^ [30] and Zr^4+^ [31,32], which have radii similar to Li^+^, helps minimize nickel–lithium disorder. During charge–discharge cycles, doping elements behave as inert species, offering interlayer support and maintaining the layered structure’s stability during deep cycling. Doping with Al^3+^ [33], Ti^4+^ [34,35], Mo^6+^ [36], and Nb^5+^ [37] enhances the layered structure, mitigating Ni^2+^ disorder. By doping elements into the transition metal layer, stronger M–O bonds are formed, which suppresses oxygen release and enhances the reversibility of the H2–H3 phase transition. Element doping, including Ta^5+^ [38], B^3+^ [39], and Nb^5+^ [40], can improve the morphology of primary particles and refine them, thus minimizing the internal stress during charge–discharge cycles and mitigating the formation of microcracks in secondary particles.

Most studies have focused on a voltage range of 2.7 V to 4.3 V, leaving limited understanding of how high-nickel NCM materials behave at higher voltage levels. This study explores the electrochemical properties of a composite material, LiNi_0.88_Co_0.05_Mn_0.07_O_2_, with niobium incorporated through bulk doping. The material is tested within a voltage range of 2.7 V to 4.5 V. This study reveals that Nb doping significantly enhances the cycling stability of high-nickel materials at elevated voltages. This advancement addresses the challenges associated with unstable high-nickel ternary material operation at high voltages, facilitating smoother performance under such conditions. The Nb-doped samples exhibit superior performance at high voltages, exhibiting a higher capacity retention rate and improved cycling performance. This offers a valuable reference for developing high-nickel ternary materials with higher energy density.

## 2. Experimental Component

### 2.1. Material Preparation

The precursor P-NCM88, with a stoichiometric ratio of Ni:Co:Mn at 0.88:0.05:0.07, was synthesized using the co-precipitation method described by Liu [41], except with the following adjustments. The Li(Ni_0.88_Co_0.05_Mn_0.07_)_1−x_Nb_x_O_2_ materials were prepared by thoroughly mixing LiOH·H_2_O, Ni_0.88_Co_0.05_Mn_0.07_(OH)_2_, and Nb_2_O_5_ in a ball mill for 10 min to achieve a uniform mixture. After grinding, the mixture was calcined at 550 °C for 6 h in an air atmosphere. Subsequently, it was further calcined at 760 °C for 15 h in pure oxygen to ensure complete doping of niobium and completion of the reaction. The molar ratio of LiOH·H_2_O to P-NCM88 to Nb_2_O_5_ was set at 1.03:(1–2x):2x.The doping level of Nb_2_O_5_ was set at 2x, with the values ranging from 0% to 1%. The Nb-doped Li(Ni_0.88_Co_0.05_Mn_0.07_)_1−x_Nb_x_O_2_ materials with doping levels of 2x = 0%, 0.3%, 0.5%, and 1% were designated as NCM88-0.3Nb, NCM88-0.5Nb, and NCM88-1Nb, respectively.

### 2.2. Material Characterization

The particle morphology of the calcined powder was examined using a Scanning Electron Microscope (SEM, SUPRA 55, Zeiss, Oberkochen, Germany). The cross-sectional SEM characterization of the ion-milled cross-sections was conducted following polishing using a cross-section polisher (I9520 CP). The crystal structure was analyzed using an X-ray Diffraction (XRD) diffractometer (Empyrean Diffractometer, Malvern Panalytical, AlmeloThe, Netherlands) equipped with a copper target Kα X-ray source. The scan was conducted within the range of 10° to 80° at a scanning rate of 5°/min. The elemental species and distribution were identified using Energy Dispersive X-ray Spectroscopy (EDS, SUPRA 55, Zeiss, Oberkochen, Germany). The material properties were characterized using Differential Scanning Calorimetry (DSC, NETZSCH, Selb, Germany), specifically to analyze the heat released during the side reactions between the cathode electrode material and the electrolyte upon completion of charging.

### 2.3. Electrochemical Testing

The button half-cells were assembled and tested in a controlled laboratory environment. The active material, acetylene black as the conductive material, and polyvinylidene fluoride (PVDF) were dissolved in N-methyl-2-pyrrolidone at a mass ratio of 8:1:1. This mixture was thoroughly agitated to form a uniform slurry. The slurry was uniformly coated onto aluminum foil and subsequently placed in a vacuum-drying oven, where it underwent drying at 110 °C for a duration of 12 h. Following drying, the electrode sheets were precisely cut into 12 mm circular discs using a slicing machine, resulting in an active material loading of 9 mg·cm^−2^. Subsequently, the button cells were assembled in a glovebox, purged with argon to ensure a controlled atmosphere. The anode electrode consisted of lithium metal foil, while the separator was a polypropylene microporous membrane. The electrolyte utilized was a 1 mol·L^−1^ LiPF_6_ solution, formulated with a 1:1:1 ratio of EC:DMC:EMC. The LAND testing system (CT3002A, Wuhan Land Electronics, Wuhan City, China) was utilized to conduct the electrochemical performance testing within the voltage range of 2.7 V–4.5 V. The specific testing procedure involved initial activation at 0.1C for three cycles, followed by constant current charging and discharging. The cycle test itself was conducted at a rate of 1C. Following activation, the multiplier test involved charging at a rate of 0.5C, followed by five cycles of charging and discharging at rates of 0.1C, 0.2C, 0.5C, 1C, 2C, 5C, and 10C, respectively, for comprehensive testing. The CV and EIS measurements were conducted on the electrochemical workstation (Zahner IM6, Zahner, Kronach, Germany). The CV testing encompassed a voltage scan ranging from 2.7 V to 4.5 V, with a scan rate of 0.1 mV·s^−1^. Additionally, the EIS experiments encompassed a frequency range spanning from 10 mHz to 1 MHz, utilizing a disturbance amplitude of 5 mV.

## 3. Results and Discussion

### 3.1. LiNi_0.88_Co_0.05_Mn_0.07_O_2_@Nb_2_O_5_ Physical Characterization

A significant body of early research has confirmed that incorporating metal cations into the heart of high-nickel ternary layered oxide cathode materials serves as a ubiquitous means of enhancement. The incorporation of metal cations significantly boosts the structural stability throughout extended charge–discharge cycles. Additionally, certain metal cations can efficiently alleviate cation intermixing between Li^+^ and Ni^2+^. Nb^5+^, as a high-valence metal, has been observed to form stronger Nb-O bonds compared to Mn–O bonds. The introduction of robust Nb-O bonds into the TM layer of high-nickel ternary cathode materials can prevent structural collapse during charge–discharge cycles, enhancing the material’s crystal structure, minimizing polarization, and ultimately bolstering its overall structural stability. Despite experiencing significant deintercalation of Li^+^ under high-voltage conditions, the material maintains its structural stability, thereby guaranteeing improved stability [42].

**Figure 1:**
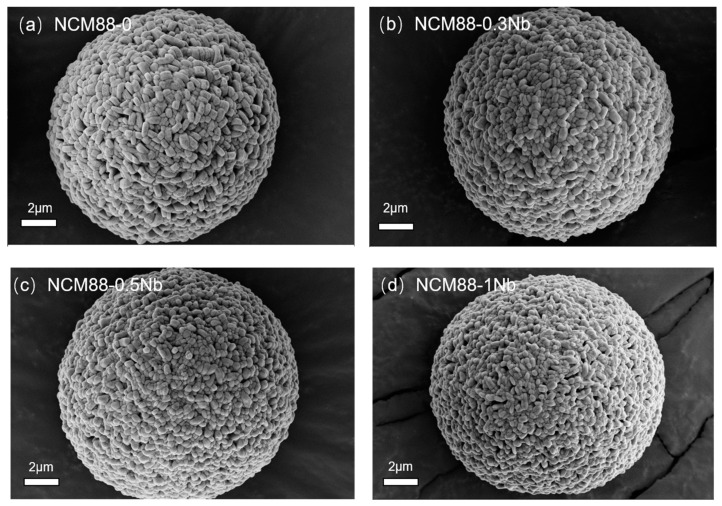
SEM images of (**a**) NCM88-0 (**b**), NCM88-0.3Nb (**c**), NCM88-0.5Nb and (**d**), NCM88-1Nb.

Figure 1 displays SEM images of undoped NCM88and Nb^5+^-doped NCM88. Across all the samples, the secondary particles consist of spherical aggregations composed of primary particles measuring several hundred nanometers, ranging in size from 12 to 15 μm. By comparing the primary particles depicted in the images, it is evident that an elevation in the Nb-doping level results in a substantial decrease in the primary particle size. Additionally, the secondary particles, which are formed through the aggregation of the primary particles, possess a more densely packed structure. The dense packing of the primary particles effectively restricts the ingress of electrolytes into the interior of the secondary particles, subsequently mitigating side reactions and bolstering the battery’s cyclic stability.

**Figure 2:**
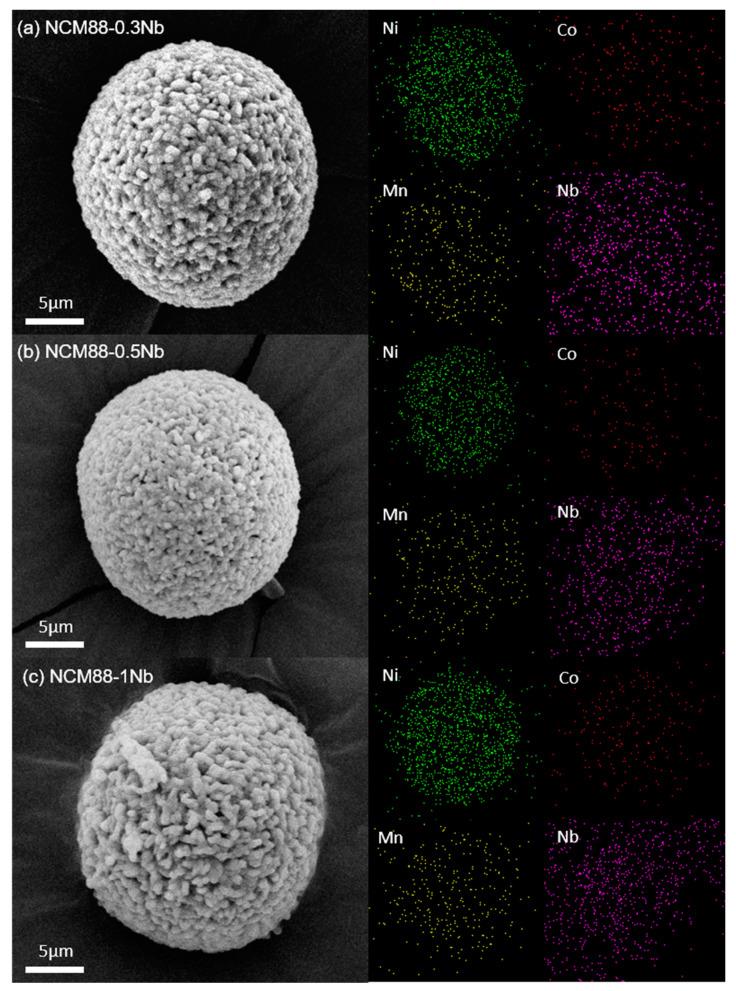
EDS-mapping image of Nb-doped samples: (**a**) NCM88-0.3Nb, (**b**) NCM88-0.5 Nb, and (**c**) NCM88-1Nb.

**Figure 3:**
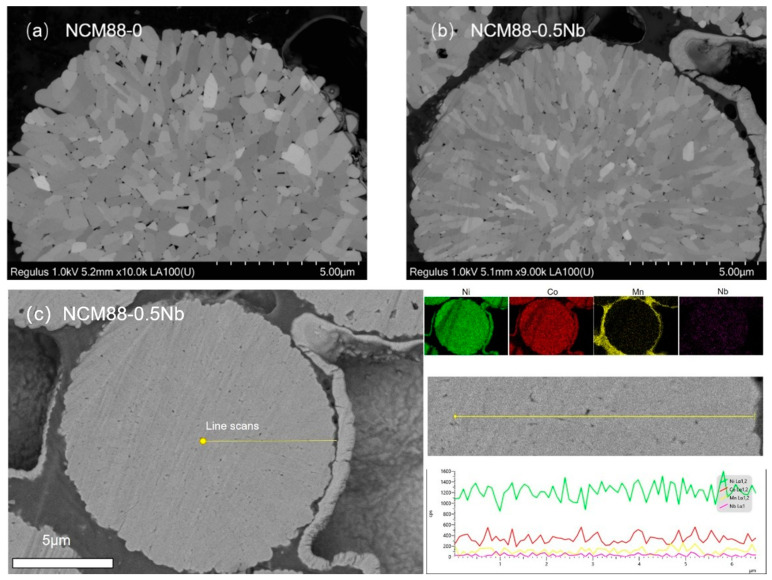
SEM images of NCM88 cathode material after ion milling: (**a**) NCM88-0, (**b**) NCM88-0.5Nb, and (**c**) EDS of NCM88-0.5 Nb.

The elemental-mapping results verify the homogeneous element distribution of Ni, Co, Mn and Nb in the Nb-doped NCM88 material (Figure 2a–c). Characterization using ion-milled SEM of the NCM88-0 and NCM88-0.5Nb materials provides valuable insights into their distinct internal microstructures. Figure 3a depicts the cross-sectional view of NCM88-0, revealing a structure composed primarily of large, irregular primary particles with intervening gaps. In contrast, Figure 3b exhibits the cross-sectional SEM image of NCM88-0.5Nb after Nb^5+^ doping, disclosing a radially arranged structure of elongated primary particles.

Prolonged charge–discharge cycling renders the NCM88-0 material susceptible to cracks due to the presence of irregular microstructures. The presence of these cracks facilitates electrolyte penetration into the cathode, leading to higher side reactions and diminished electrochemical performance. Conversely, NCM88-0.5Nb’s secondary particles exhibit radially aligned rod-like structures. This morphology mitigates mechanical strain, prevents intergranular cracking, minimizes electrolyte contact, and enhances cycling stability under high-voltage conditions [43]. Characterization of the high-nickel ternary cathode materials via EDS reveals the peak appearance and intensity, indicating the presence and concentration of specific elements. Analysis of these peaks provides insights into the distribution and relative concentrations of key elements within the material. Quantification of the excess metals in an EDS line scan is achieved by analyzing the data fluctuations. In the NCM88-0.5Nb sample, the nickel content is elevated, resulting in prominent peaks, while the cobalt and manganese peaks share similar features. Although the niobium peaks are relatively low, they are uniformly distributed. The characterization results confirm the presence and uniform distribution of the four elements: Ni, Co, Mn, and Nb.

**Figure 4:**
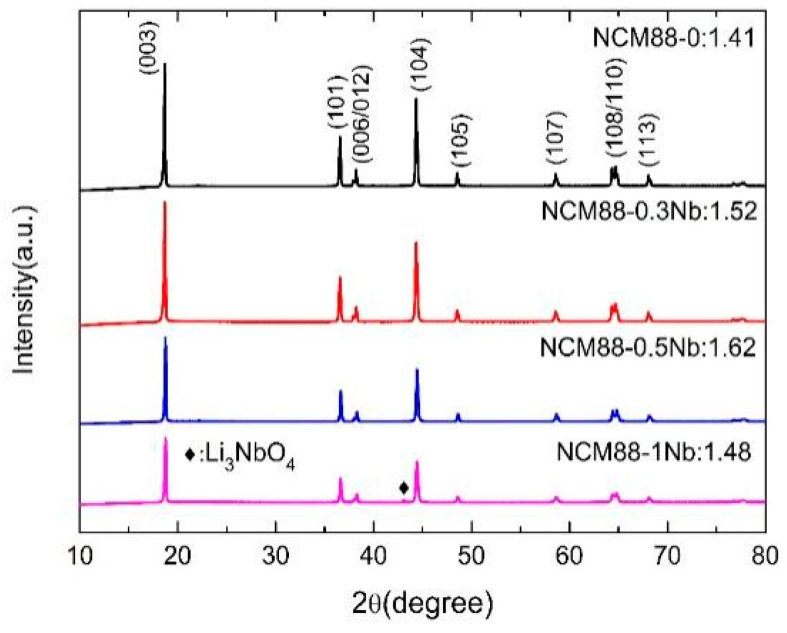
XRD pattern ofNCM88-0, NCM88-0.3Nb, NCM88-0.5Nb and NCM88-1Nb.

We conducted XRD characterization tests on the NCM88-0, NCM88-0.3Nb, NCM88-0.5Nb, and NCM88-1Nb samples to investigate the influence of Nb doping on the crystal structure of NCM88 materials. Figure 4 clearly demonstrates that the diffraction peaks of NCM88-0, NCM88-0.3Nb, and NCM88-0.5Nb are identical, indicating that Nb doping does not introduce additional impurity peaks. This confirms that the successful integration of Nb doping into the NCM88 lattice does not alter its crystal structure. The XRD pattern of NCM88-1Nb, at a doping level of 1%, exhibits impurity peaks attributed to Li_3_(NbO_4_) (PDF#82-1198), primarily due to the interaction between the excess Nb_2_O_5_ and surface LiOH [44]. The sharpness of all the diffraction peaks is indicative of the excellent crystallinity of the prepared materials, exhibiting a layered structure belonging to the hexagonal R-3m space group of α-NaFeO_2_. Lithium atoms occupy the 3a position, transition metals occupy the 3b position, and oxygen atoms reside in the 6c position. Notably, the observed splitting of the I(006)/I(102) and I(018)/I(110) peaks indicates a highly ordered layered structure in the prepared materials. The comparison of the R[I(003)/I(104)] values, exceeding 1.2 for all the materials, indicates low cation mixing within the materials.

As the concentration of the Nb doping increases, the primary factor that reduces cation mixing is the exchange of Nb^5+^ ions with Li^+^ ions during the heat treatment process. This interaction takes place within the lithium layer, leading to a high cathode charge that effectively repels Ni^2+^ ions back to the transition metal layer. Excessive cation mixing and discharging result in the degradation of electrochemical performance, a reduction in structural stability, and compromised safety performance. The occupation of Nb elements within the crystal lattice, along with the matching of ionic radii, effectively mitigates lithium–nickel mixing and discharging. Consequently, this enhances the crystal structure and minimizes the structural changes and lattice distortions during charge–discharge cycles, ultimately enhancing the cycling stability of the material at elevated voltages.

The Rietveld refinement of the XRD data is further detailed in Table 1. The consistently high values of c/a, exceeding 4.9, suggest an outstanding layered structure for the material. As the doping of Nb increases, all the lattice parameters (a, c) and the unit volume display a corresponding increase. This is attributed to the larger ionic radius of Nb^5+^ (0.64 A) compared to Ni^3+^ (0.56 A), Co^3+^ (0.545 A), and Mn^4+^ (0.53 A); however, it is still smaller than Li^+^ (0.76 A). The incorporation of Nb^5+^ via doping replaces the original transition metal ions, subsequently resulting in an expansion of the material’s lattice parameters. Given the significantly higher bond energy of Nb-O (ΔHf298(Nb-O) = 753 kJ mol^−1^) compared to Li-O (ΔHf298(Li-O) = 341 kJ mol^−1^), the introduction of the stronger Nb-O bond enhances the stability of the material’s structure. The integration of Nb^5+^ within the Li layer enhances the cycling stability of charge and discharge over extended periods, ultimately leading to the improved electrochemical performance of the material, as reported in reference [45]. The value of the c-axis, which is crucial for Li^+^ migration, increases as the doping level increases. The introduction of Nb^5+^ modifies the lithium-ion diffusion pathway within the material, resulting in a smoother trajectory. By mitigating the diffusion resistance, the material’s discharge capacity and rate performance are improved. Furthermore, it facilitates the reduction of the volume fluctuations during charge–discharge cycles. Consequently, this minimizes internal stress accumulation and preserves structural integrity. The specific XRD refinement pattern is shown in Figure S1 in the Supporting Information.

**Figure 5:**
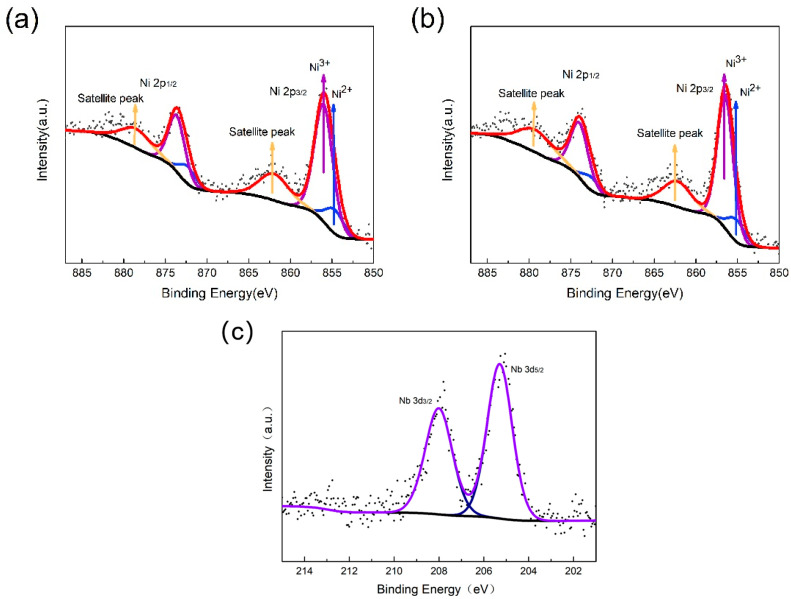
XPS profile of NCM88: (**a**) NCM88-0 and (**b**,**c**) NCM88-0.5Nb.

XPS was used to further characterize the valence state of elemental Ni. The fitted curves in Figure 5a,b were analyzed for the ionic Ni2P^3/2^ energy levels of Ni^3+^ and Ni^2+^. Specifically, for NCM88-0, the energy levels are 856.01 eV and 854.7 eV, while for NCM88-0.5Nb, they are 856.47 eV and 855.16 eV. The energy levels of Ni^3+^/Ni^2+^ and Ni2p^3/2^ are influenced by excess Li across all the samples. The fitting results indicate an increase in Ni^3+^ content and a decrease in Ni^2+^ content upon doping with Nb. Consequently, in high-nickel ternary cathode materials, Nb doping not only occupies Li sites but also hinders the diffusion of Ni^2+^ to Li^+^ sites, reducing cation mixing and enhancing the electrochemical performance of cathode materials. However, an excessive proportion of added Nb can not only hinder the diffusion of Ni^2+^ to Li^+^ sites but also accumulate on particle surfaces, thereby impeding Li^+^ transport and decreasing the charge–discharge specific capacity of the material. Therefore, an appropriate doping amount can optimize the electrochemical performance of the material. XPS analysis of the Nb element is conducted on the NCM88-0.5Nb sample, revealing binding energies of Nb 3d^3/2^ and Nb 3d^5/2^ at 205.3 eV and 208.2 eV, respectively, as depicted in Figure 5c, suggesting the presence of Nb^5+^ in the layered structure.

**Figure 6:**
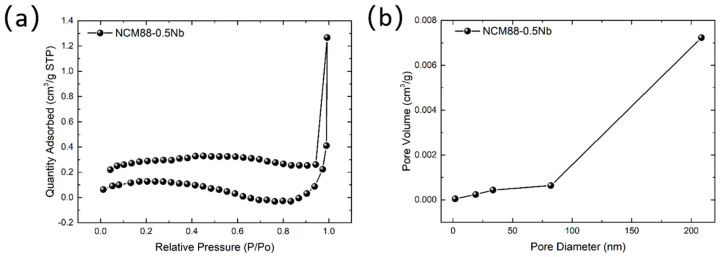
(**a**) N_2_ adsorption–desorption curve, and (**b**) pore size distribution of the NCM88-0.5Nb sample.

BET analysis was employed to determine the specific surface area of the composites, and the results are presented in Figure 6. The adsorption and desorption isotherms exhibit type II characteristics, with a distinct hysteresis loop (H3), indicating the presence of mesoporous and macroporous structures in the material. NCM88-0.5Nb exhibits a specific surface area of 0.4277 m^2^/g, a pore volume of 0.001960 cm^3^/g, and an average pore size of 18.3290 nm. The compaction of particles due to the action of Nb results in the reduced specific surface area of the composites, leading to a decrease in the area of side reactions between the active material and the electrolyte. This reduction in side reactions prolongs the electrolyte’s lifespan. A reduced specific surface area minimizes the interface for side reactions between the active material and electrolyte, ultimately prolonging the battery’s cycle life. The BJH pore size distribution diagram reveals that the composite material primarily comprises mesoporous and macroporous structures.

### 3.2. LiNi_0.88_Co_0.05_Mn_0.07_O_2_@Nb_2_O_5_ High-Voltage Electrochemical Performance Test

**Figure 7:**
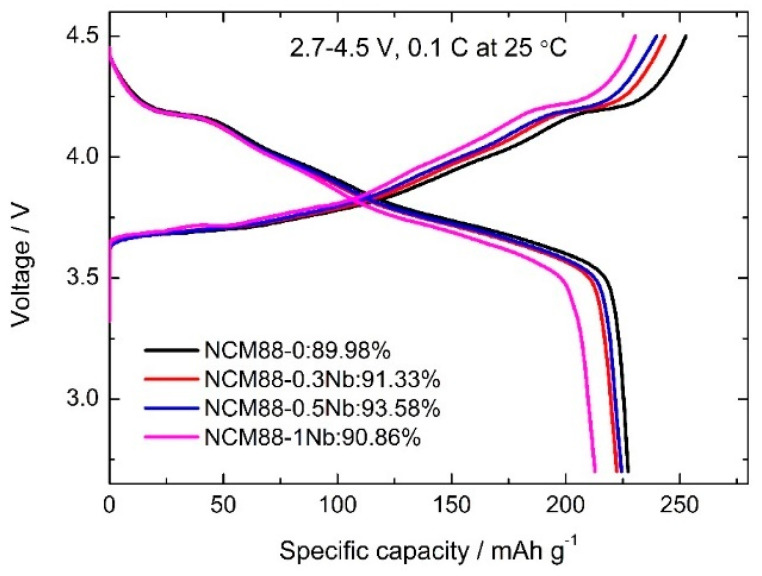
The first charge-discharge curve of NCM88 at a high cut-off voltage of 2.7 V–4.5 V.

The electrochemical performance of the cathode materials was assessed using coin-type CR2032 lithium metal button cells. The cells were activated through three cycles at a high cut-off voltage ranging from 2.7 V to 4.5 V and a current density of 0.1C (equivalent to 18 mAh g^−1^). The cycling stability of the materials at high voltage was assessed through 100 cycles at 1C (equivalent to 180 mAh g^−1^). The initial discharge specific capacities of NCM88-0, NCM88-0.3, NCM88-0.5Nb, and NCM88-1Nb at 0.1C were measured to be 227.3, 222.4, 224.4, and 212.8 mAh g^−1^, respectively. As shown in Figure 7, an increase in the Nb^5+^ doping level results in a decrease in the discharge-specific capacity of the materials. The decline in discharge capacity becomes more pronounced at a doping level of 1%, possibly attributed to the reaction between the excess Nb_2_O_5_ and surface LiOH, leading to the formation of Li_3_NbO_4_ and a consequent reduction in the electrochemical performance of the material.

**Figure 8:**
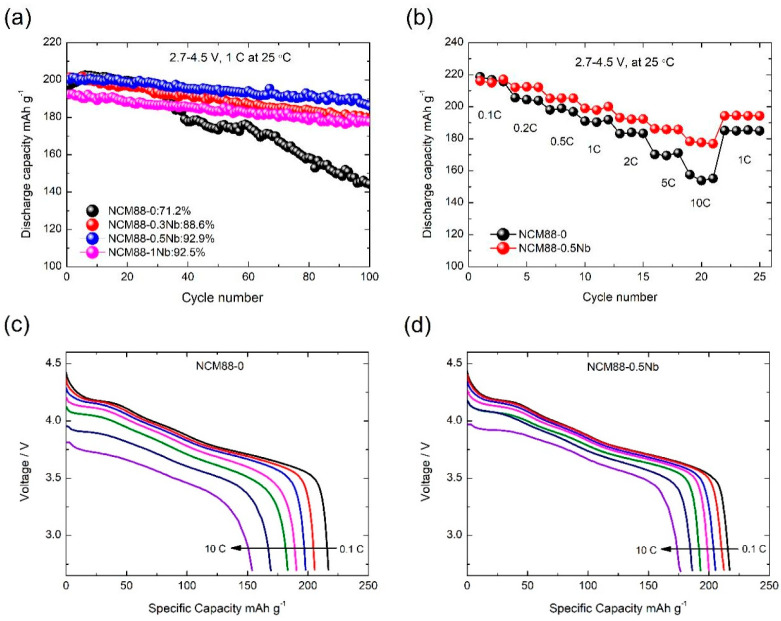
Electrochemical performance of NCM88 at high cut-off voltages of 2.7 V–4.5 V: (**a**) cycle performance of NCM88, (**b**) rate performance of NCM88, (**c**) capacity distribution of NCM88-0 at different magnifications, and (**d**) capacity distribution of NCM88-0.5Nb at different magnifications.

The materials were tested at a temperature of 25 °C and a voltage range of 2.7 V–4.5 V, undergoing 100 cycles at a 1C rate. This was performed to assess their cycling stability under high-voltage conditions. The test data, presented in Figure 8, visually demonstrate the cycling stability results. The initial discharge specific capacities of NCM88-0, NCM88-0.3Nb, NCM88-0.5Nb, and NCM88-1Nb, measured during the initial discharge cycle, were 197.2, 201.5, 200.3, and 192.4 mAh g^−1^, respectively. Following 100 charge–discharge cycles, the capacity retention rates for NCM88-0, NCM88-0.3Nb, NCM88-0.5Nb, and NCM88-1Nb are 71.2%, 88.6%, 92.9%, and 92.5%, respectively, indicating their cycling stability at high voltages. As the doping level of Nb^5+^ increases, a slight enhancement in the initial discharge specific capacity of the materials is observed, along with a notable improvement in the cycling retention rate.

At a doping level of 1%, a slight capacity decrease is noted. This can be attributed to the surface formation of Li_3_NbO_4_, resulting from an excess of Nb_2_O_5_. This, in turn, hinders the transport of Li^+^ ions, leading to a subsequent capacity loss. The increased cut-off voltage places stringent demands on the performance of the cathode materials. Despite these stringent demands, Nb-doped high-nickel ternary cathode materials exhibit remarkable performance. The 0.5% Nb-doped sample exhibits an impressive capacity retention of 92.9%, significantly surpassing the undoped sample’s performance of 71.2%. This underscores the superior structural stability and electrochemical performance of Nb-doped cathode materials, particularly under high-voltage conditions.

Additionally, tests were performed on the materials at 25 °C, utilizing a cut-off voltage ranging from 2.7 V to 4.5 V, and a charging rate of 0.5C. The specific discharge capacity was measured across multiple discharge rates, including 0.1C, 0.2C, 0.5C, 1C, 2C, 5C, and 10C. For NCM88-0, the average discharge specific capacity is 217.1 mAh g^−1^ at 0.1C and 155.5 mAh g^−1^ at 10C, resulting in a capacity retention of approximately 71.64%. NCM88-0.5Nb demonstrates an average discharge capacity of 216.1 mAh g^−1^ at 0.1C and 177.6 mAh g^−1^ at 10C, maintaining a capacity retention of 82.21%. Following cycling at various rates, NCM88-0 exhibits a capacity retention rate of 96.8% at 1C, compared to NCM88-0.5 Nb’s rate of 97.7%. The rate tests demonstrate that at a low rate of 0.1C, the average discharge specific capacities of NCM88-0 and NCM88-0.5Nb are comparable. At a higher rate of 10C, NCM88-0.5Nb demonstrates a notably superior capacity compared to NCM88-0. This improvement is attributed to the enhancement of the Li^+^ ion transport pathways through the introduction of Nb^5+^ doping. The increased capacity retention rate is attributed to the doping of Nb^5+^ within the lattice structure, which enhances the material’s stability during high-rate charge–discharge cycles, thereby maintaining structural integrity during rapid lithium extraction. When compared to other NCM cathode materials, as detailed in Table 2, the electrochemical performance of our material demonstrates promising outcomes.

**Figure 9:**
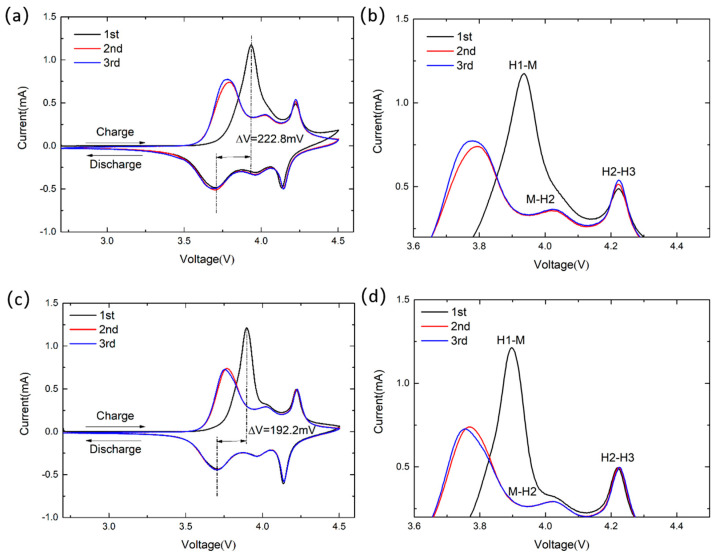
CV curves for the first three turns at a cut-off voltage of 2.7 V–4.5 V: (**a**,**b**) NCM88-0 and (**c**,**d**) NCM88-0.5Nb.

Cyclic voltammetry (CV) tests were conducted on the materials within the voltage range of 2.7 V to 4.5 V. The results are presented in Figure 9a–d. The three pairs of redox peaks observed in Figure 9a,b correspond to distinct phase transitions of lithium ions during detachment and embedding processes. The formation of the larger oxidation peaks during the first cycle is primarily attributed to the decomposition of the electrolyte, resulting in the deposition of a cathode material layer on the CEI film surface. During charging, NCM88-0 exhibits a prominent oxidation peak at approximately 3.93 V (Figure 9a), accompanied by two smaller peaks at 4.02 V and 4.22 V. The oxidation peaks primarily result from the formation of a CEI film on the cathode material surface following the electrolyte decomposition. The larger oxidation peak is primarily attributed to the oxidation of Ni^2+^ to Ni^3+^ and Ni^4+^, leading to a material transition from the hexagonal phase (H1) to the monoclinic phase (M). In contrast, the smaller peaks are associated with transitions from the monoclinic phase (M) to the hexagonal phase (H2) and from the hexagonal phase (H2) to the hexagonal phase (H3). The potential difference (ΔV) between the oxidation and reduction peaks exhibiting the greatest contrast is potentially due to the formation of a CEI film on the cathode material surface. Specifically, the ΔV for NCM88-0 is 222.8 mV, whereas that for NCM88-0.5Nb is approximately 192.2 mV. The doping of Nb^5+^ effectively mitigates the potential difference enhancement, thereby improving the degree of polarization during battery charging and discharging. Figure 9b,d correspond to magnified views of Figure 9a,c, respectively. The intensity of the oxidation peak of NCM88-0, located near 4.22 V, decreases during cycling, indicating that the emergence of the hexagonal phase (H3) contributes to a gradual capacity degradation. For NCM88-0.5Nb, there is an overlap near 4.22 V, resulting in a relatively stable peak intensity. This observation suggests that the doping of Nb^5+^ mitigates the phase transition from H2 to H3, thereby enhancing the cycling reversibility of the material.

**Figure 10:**
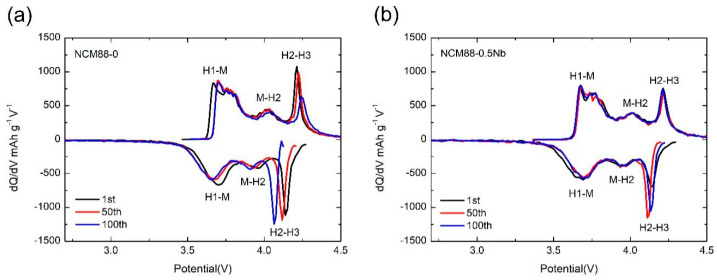
dQ dV^−1^-V curves for the 1st, 50th, and 100th cycles: (**a**) NCM88-0, and (**b**) NCM88-0.5Nb.

Figure 10 provides additional evidence of the improved structural stability of NCM88-0.5Nb compared to NCM88-0 through the creation of a dQ/dV^−1^-V plot. Figure 10b shows the dQ/dV^−1^ plots for the initial, 50th, and 100th charge–discharge cycles of NCM88-0 and NCM88-0.5Nb under conditions of 25 °C and a cut-off voltage range of 2.7 V–4.5 V. The prominent peaks observed in Figure 10b indicate phase transitions within the cathode material during multiple charge–discharge cycles. Figure 10a reveals pronounced alterations in the H2–H3 phase transition of NCM88-0 upon exposure to high-voltage cycling, suggestive of irreversible structural degradation in the cathode material. The predominant cause underlying these observations is the intensified anisotropic volume changes at 4.5 V, leading to the propagation of microcracks or fractures within the material particles. Consequently, this leads to increased material resistance, accelerated capacity degradation during cycling, and compromised electrochemical performance. By contrast, Figure 10b demonstrates that for NCM88-0.5Nb, there is minimal displacement of the H2–H3 phase transition in the dQ/dV^−1^ curve, even as the cycle count increases, suggestive of the excellent reversibility of the phase transitions. This observation is primarily ascribed to the Nb^5+^ doping, which reduces the lattice parameters and effectively mitigates structural collapse induced by deep discharge at high voltages. Additionally, the Nb^5+^ doping modifies the morphology of the primary particles, resulting in the development of elongated rod-like structures exhibiting a radial distribution. The noted alterations in the microscopic structure effectively hinder anisotropic volume changes at high voltages, subsequently minimizing the microcracks and particle fractures that may occur during extended charge–discharge cycles. This, in turn, serves to further augment the electrochemical performance of the material under high-voltage conditions.

**Figure 11:**
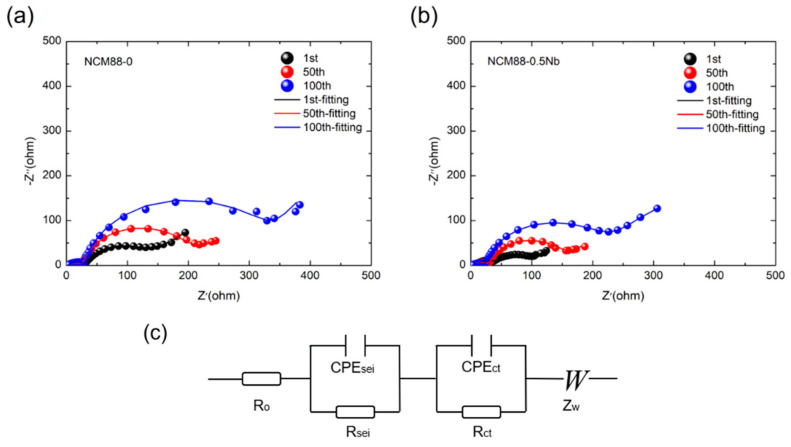
Electrochemical impedance fitting of NCM88 after 1, 50, and 100 cycles at a high cut-off voltage of 2.7 V–4.5 V: (**a**) NCM88-0, (**b**) NCM88-0.5Nb, and (**c**) analog circuit diagram.

We employed EIS testing to assess the impact of Nb^5+^ doping on the electrochemical performance and kinetics of high-nickel ternary cathode materials. The battery, charged to 4.5 V, underwent testing during the 1st, 50th, and 100th cycles. Figure 11 depicts the EIS spectra of NCM88-0 and NCM88-0.5Nb at a rate of 1C, within the voltage range of 2.7 V to 4.5 V at 25 °C. The spectra exhibited two distinct semi-circles in the high-and mid-frequency regions, along with a linear feature in the low-frequency region. The intersection of the highest-frequency semicircle with the real axis signifies the cell’s Ohmic resistance (R_o_), whereas the high-frequency semicircle corresponds to the resistance associated with Li^+^ passing through the SEI membrane. The mid-frequency region is associated with charge transfer resistance, and the low-frequency region relates to Li^+^ diffusion within active material particles. The EIS spectra were fitted based on the equivalent circuit diagram depicted in Figure 11. Table 3 presents the fitting outcomes for the Ohmic resistance (R_o_), SEI membrane resistance (R_SEI_), and charge transfer resistance (R_ct_). Across the 1st, 50th, and 100th cycles, the Ohmic resistance (R_o_) of all the samples remains negligibly low, falling below 3 Ω. The initial charge transfer resistance (R_ct_) of NCM88-0 is 80.03 Ω, increasing to 154.22 Ω after 50 cycles, and ultimately reaching 292.2 Ω after 100 cycles. This indicates that numerous charge–discharge cycles cause surface damage to the material, leading to an elevation in charge transfer resistance. Following Nb^5+^ doping, the R_ct_ of NCM88-0.5Nb exhibits a significant reduction throughout the 1st, 50th, and 100th cycles. This notable decrease, representing approximately a 41.9% reduction after 100 cycles, underscores the efficacy of Nb^5+^ doping in minimizing charge transfer resistance and subsequently improving interface stability. The introduction of Nb^5+^ doping alters the inherently disordered microstructure of the cathode material, transforming it into radially oriented rod-like structures. This configuration effectively prevents electrolyte infiltration, minimizes side reactions, reduces overall material resistance, and enhances cycling stability and rate capability.

**Figure 12:**
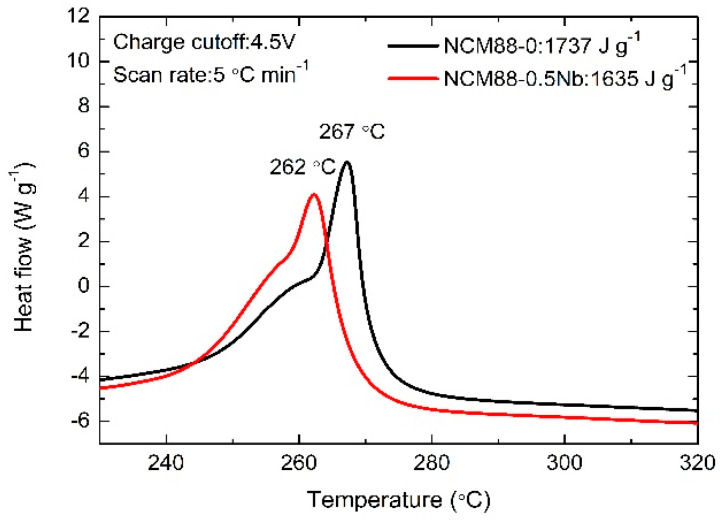
DSC plots of NCM88-0 and NCM88-0.5Nb at high cut-off voltages of 2.7 V–4.5 V.

Figure 12 presents the DSC analysis results for the NCM88-0 and NCM88-0.5Nb electrodes, both charged to 4.5 V. The analysis was performed in a 1.2 M LiPF_6_ electrolyte based on carbonate, including EC and EMC. The objective of this analysis was to explore the side reactions occurring between the cathode electrode material and the electrolyte in a fully charged condition (i.e., highly de-lithiated state). Both the NCM88-0 and NCM88-0.5Nb cathode electrodes exhibit prominent exothermic peaks, specifically at 267 °C and 262 °C, respectively. These peaks correspond to heat releases of 1737 J g^−1^ and 1635 J g^−1^, respectively. The inclusion of Nb^5+^ doping results in a reduction in the heat released by the material and a 5 °C delay in the characteristic peak of the exothermic reaction, as documented in reference [50]. Subsequently, the integration of Nb^5+^ doping in NCM88 leads to the formation of distinctly elongated primary particles with a radial distribution. This effectively minimizes the isotropic volume changes, prevents microcracks from forming on the secondary particle surfaces, limits electrolyte infiltration into the cathode electrode material, and enhances the thermal properties of the cathode electrode material. The decrease in thermal stability serves as evidence that Nb^5+^ doping within the lattice parameters of NCM88 enhances the material’s stability. By optimizing the material’s microstructure, side reactions are further minimized, effectively mitigating the safety risks associated with intense interactions between the cathode electrode material and the electrolyte.

### 3.3. Surface and Cross-Sectional Morphological Characterization of LiNi_0.88_Co_0.05_Mn_0.07_O_2_@Nb_2_O_5_ after Cycling

**Figure 13:**
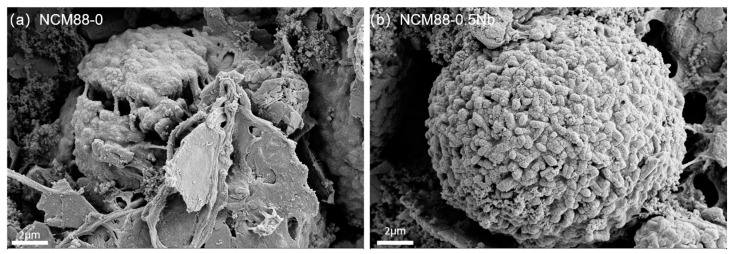
SEM image after 100 charge–discharge cycles at a cut-off voltage of 2.7 V–4.5 V. (**a**) NCM88-0 (**b**) NCM88-0.5Nb.

SEM analysis of the cathode material reveals cracks on the surface of the NCM88-0 secondary particles following 100 cycles at 1C between 2.7 V and 4.5 V, as seen in Figure 13a. These cracks allow the electrolyte to penetrate the material, leading to a reactive Ni^4+^ surface formation of NiO, which detracts from the material’s capacity. In contrast, the surface of the NCM88-0.5Nb maintains an intact secondary particle structure, as shown in Figure 13b. This configuration effectively prevents electrolyte infiltration and minimizes phase transformation. The enhanced electrochemical properties of these materials primarily result from the Nb^5+^ doping, altering the cathode’s microstructure. The slender rod-like structure distributed radially effectively mitigates anisotropic volume changes during cycling, preventing irreversible phase transformations and bolstering cyclic stability.

**Figure 14:**
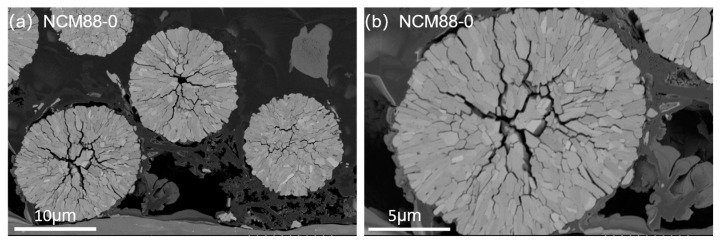

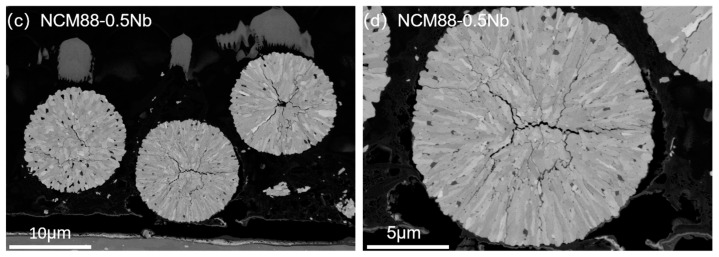
SEM image of NCM88 after 100 cycles at a high cut-off voltage of 2.7 V–4.5 V: (**a**,**b**) NCM88-0 and (**c**,**d**) NCM88-0.5Nb.

Following 100 cycles, SEM analysis using ion milling at a discharge rate of 1C and a high cut-off voltage ranging from 2.7 V to 4.5 V reveals pronounced disparities in the surface morphology of the cathode electrode material between NCM88-0 and NCM88-0.5Nb. This is illustrated in Figure 14a,b. Upon completion of 100 cycles, the NCM88-0 secondary particles remain intact without visible fractures, whereas the NCM88-0.5Nb particles exhibit subtle cracks, preserving their structural integrity [51]. This is primarily attributed to the doping of Nb^5+^, which modifies the irregular aggregation of the original primary particles, resulting in radially distributed elongated rod-like structures. This structural modification effectively mitigates the significant anisotropic volume changes during high-voltage cycling, subsequently improving the material’s cycling stability. Additionally, during deep discharge conditions at high voltages, the introduction of Nb-O bonding into the lattice mitigates structural collapse, facilitating the transport of Li^+^ ions. This ensures the material’s superior electrochemical performance, even under high-voltage conditions, thereby enhancing its cycling stability.

## 4. Discussion

A high-nickel ternary cathode electrode material, LiNi_0.88_Co_0.05_Mn_0.07_O_2_, doped with Nb^5+^ was successfully synthesized using the high-temperature solid-phase method. Its electrochemical performance was evaluated at high voltages. The research results indicate that, at a 1C rate and a cut-off voltage range of 2.7 V–4.5 V, the cyclic retention rate of the material doped with 0.5% Nb^5+^ is significantly higher, reaching 92.9% compared to the undoped material’s 71.2%. After high-rate cycling at 10C and returning to 1C, the material exhibits a capacity retention rate of 97.7%. The composite material prepared demonstrates excellent cyclic stability and capacity retention, particularly at high rates and voltages. This enhanced performance is primarily attributed to the Nb^5+^ doping, which modifies the morphology of the primary crystals, resulting in a radially distributed, elongated rod-like structure. This unique structure effectively mitigates microcrack formation during deep charge–discharge cycles, further contributing to its excellent cyclic stability. The formation of strong Nb-O bonds prevents oxygen precipitation and mitigates irreversible phase transitions at high voltages, thereby improving the material’s electrochemical performance.

By analyzing the dQ/dV^−1^-V curves, it is evident that the material’s polarization decreases significantly upon Nb^5+^ doping compared to the undoped material. This suggests that the incorporation of Nb^5+^ effectively hinders the H2 to H3 phase transition in the material. Upon comparing the EIS spectra, it is evident that the material’s resistance exhibits significant changes as the number of cycles increases. Following 100 cycles at a high cut-off voltage ranging from 2.7 V to 4.5 V, the charge transfer impedance for NCM88-0.5Nb and NCM88-0 is measured to be 170.27 Ω and 292.9 Ω, respectively. This indicates that Nb^5+^ doping effectively mitigates structural changes in the material, leading to smaller charge transfer impedances following repeated charge–discharge cycles. The effective mitigation of structural changes in the material through Nb^5+^ doping results in smaller charge transfer impedances after numerous charge–discharge cycles, significantly enhancing the material’s electrochemical performance at elevated voltages.

## Figures and Tables

**Table 1:** Lattice parameters of NCM88-0, NCM88-0.3Nb, NCM88-0.5Nb and NCM88-1Nb after refinement.

Sample	a (A)	c (A)	c/a	V (A3)	R (I003/I104)	Rwp (%)
NCM88-0	2.88372	14.2388	4.937651	102.444	1.41	3.1
NCM88-0.3Nb	2.88411	14.2395	4.937218	102.474	1.52	2.14
NCM88-0.5Nb	2.88423	14.2428	4.938179	102.539	1.68	2.28
NCM88-1Nb	2.88517	14.2493	4.938808	102.704	1.48	2.76

**Table 2:** Comparison of the results of the electrochemical performance of other cathode materials at high voltages with the results of this study.

Samples	Voltage Range	Discharge Capacity	Rate/Capacity Retentions/ Cycle Number	Reference
LiNi_0.88_Co_0.05_Mn_0.07_O_2_@Nb_2_O_5_	2.7 V–4.5 V	200.3/1C	1C/92.9%/100	This work
NCM811-PFPN	3.0 V–4.5 V	187.6/1C	1C/89.5%/200	[46]
SC-NCM811 (Ce/Al doping)	2.8 V–4.5 V	188.5/1C	1C/85.38%/100	[33]
SC-NCM811 (Y doping)	2.7 V–4.5 V	189.5/1C	1C/94.53%/100	[47]
NCM811@MTP	2.7 V–4.5 V	201.5/1C	1C/89.3%/200	[48]
NCM8155	2.7 V–4.5 V	202.1/1C	1C/88.02%/100	[49]

**Table 3:** The R_o_, R_SEI_ and R_ct_ values of NCM88-0 and NCM88-0.5Nb after fitting.

Sample	NCM88-0			NCM88-0.5Nb		
	R_o_ (Ω)	R_SEI_ (Ω)	R_ct_ (Ω)	R_o_ (Ω)	R_SEI_ (Ω)	R_ct_ (Ω)
Cycling						
1st	2.206	28.915	80.03	2.2996	52.769	37.646
50th	2.468	27.803	154.22	2.4016	28.147	109.11
100th	2.588	23.363	292.9	2.752	20.909	170.27

## Data Availability

No new data were created or analyzed in this study. Data sharing is not applicable to this article.

## Supplementary Materials

The following supporting information can be downloaded at: https://www.mdpi.com/article/10.3390/ma17092127/s1, Figure S1: XRD Rietveld refinement patterns of (a) NCM88-0 (b), NCM88-0.3Nb (c) NCM88-0.5 Nb (d) NCM88-1Nb.
