# Supplementary material for: A Study on the Microstructure Regulation Effect of Niobium Doping on LiNi0.88Co0.05Mn0.07O2 and the Electrochemical Performance of the Composite Material under High Voltage
Source: Materials (Basel). 2024 Apr 30;17(9):2127. doi: 10.3390/ma17092127 (PMC11084928; doi:10.3390/ma17092127)
Supplement: Supplementary file 1 [file materials-17-02127-s001.zip › materials-2969282-supplementary.pdf]

Supporting information

The Electrochemical Performance And Microstructure of $LiNi_{0.88}Co_{0.05}Mn_{0.07}O_2$ Cathode Material Working In High-voltage By Niobium Doping

Xinrui Xu, Junjie Liu, Bo Wang, Jiaqi Wang, Weisong Meng, Feipeng Cai*

Energy Institute, Qilu University of Technology (Shandong Academy of Sciences), Jinan, 250014, China

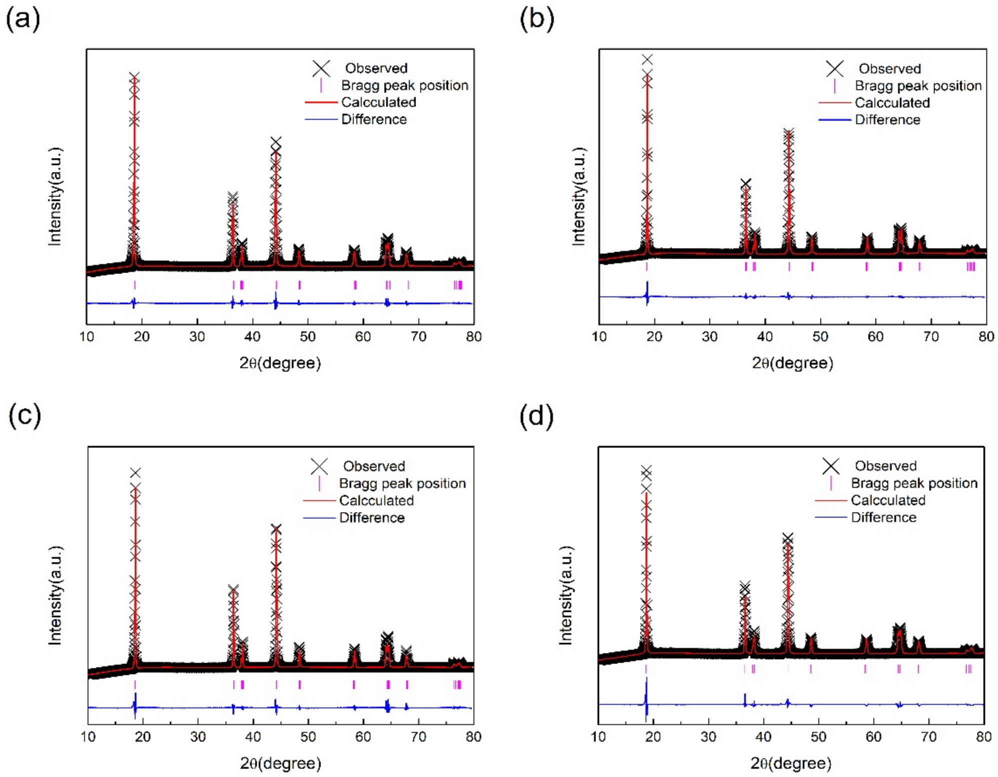

Figure S1. XRD Rietveld refinement patterns of (a) NCM88-0 (b), NCM88-0.3Nb (c) NCM88-0.5 Nb (d) NCM88-1Nb
